# Case of Tetanus from Very Slight Injury

**Published:** 1860-12

**Authors:** D. W. Hand

**Affiliations:** St. Paul, Min.


### ARTICLE 4.

### CASE OF TETANUS FROM VERY SLIGHT INJURY.

BY D. W. HAND, M. D., ST. PAUL, MIN.

November 2d, 1860, I was called, at 3 o'clock in the morning, to see Mrs. O'Bryan, living in West St. Paul, on the low bottom land bordering on the river.

Mrs. O'B. is a strong, hearty woman, aged 22, having two children, and now advanced three months in pregnancy. On Saturday, October 27th, as she was splitting kindling wood, a small piece flew up and hit her over the right eye, hurting her considerably at the time; but as it only bled a few drops, and seemed a mere scratch, she put on a piece of sticking plaster, and thought no more of it. The weather was very wet at the time, and being out of doors frequently, she supposes she took cold, for in a day or two the wound became swelled and somewhat painful. She then began to have pain in the limbs, and aching between her shoulders, but did not think herself sick until the morning of November first, when she found on awakening, a slight stiffness of her jaws, and considerable difficulty in swallowing. She also noticed that she could not close her right eye; even while sleeping it remained partially open. All that day she lay in bed, without eating or drinking anything. She passed much urine, and toward evening she had a very copious evacuation from the bowels. During the early part of the night she slept some, and noticed that several times during her sleep she bit her tongue.

About 2 o'clock she was awakened by a sensation of choking, and I was then sent for. I found her lying very comfortably on her back, her mouth drawn a little to one side, and the right upper eye-lid drawn up, so as to give her a very sinister expression. He skin was moist, but a little cold, and her pulse 77. She talked to me, through her teeth, in a voice composed and natural; complained of pain between the shoulders, and of thick phlegm in her throat, which she could neither get up or down. There was no stiffness of the neck, no disturbance of the stomach, nor had the attendant noticed anything like spasms.

The wound over her eye, which appeared to be a small punctured one, had healed, but the cicatrix was depressed and very red, and immediately above it was a small soft tumor. The massster muscles were firmly contracted; they felt hard, but were not tender to the touch. By strong efforts she could open the jaws enough to allow me to pass the handle ot a teaspoon between between the truth. I gave her a tea-spoonful of vinegar and water which seemed to clear her throat, and she then swallowed without much difficulty half a grain of morphine and an eighth of a grain of tartarized antimony in a little water.

I applied a warm poultice to the wound and also to the jaws, and directed another morphine powder to be given at 8 o'cloek; and every two hours a mixture of ext. belladonna and ext. valerian, and, finding the patient inclined to sleep, left her, at 5 A. M.

At 1 o'clock P. M. I saw her again. Found her with her head thrown back, and considerable difficulty of breathing. She was sweating freely, and her skin was warm; pulse 80, full and strong; said she felt sick at her stomach, but could not vomit; neither could she swallow a drop of anything.

She had taken the morphine powder at 8 o'clock, and after it one dose of the mixture, but could swallow no more after that. In attempting to take a little water for me, she had a partial convulsion; throwing her head back, and raising her chest and abdomen. It lasted but a few seconds, and she did not lose consciousness. Her nurse then said she had had five such spells during the forenoon.

Chloroform applied to the masseter muscles relaxed them somewhat, but did not relieve the choking in the throat. I then caused her to inhale the chloroform, and while the muscles were relaxed by it, put ten grains of calomel, with a little water, in her mouth. It was all thrown out at the corner of the mouth in a few minutes; she could swallow nothing. She had no head-ache, and the pain in her back was relieved. Bowels had not been opened, or urine passed since my former visit. Ordered a purgative enema, to be followed every two hours by an injection of assafœtida and pulv. ipecac āā ʒ p. ℥ iv warm water.

At 2 o'clock the next morning, November 2d, I was called in haste to see her; found she had just had a very severe convulsion, lasting at least five minutes. From the account of her attendants, I think she had a complete convulsion, but of shorter duration about an hour before. She was now sweating profusely and very nervous, but answered my questions rationally, said she had passed urine several times with difficulty, that she felt no pain at the moment but thought she should choke; that she could not bear the poultice on the wound as it hurt her, and that all around the swelling there was a continual itching. The tumor had increased since my last visit, and looked much inflamed. She would not let me touch it. I administered chloroform and while she was under its influence I passed a lancet through the tumor. Just beneath the skin appeared a mass of clotted blood and from beneath it came two or three drachms of venous blood. A few drops of trickling down her cheek, as the effect of the chloroform passed away, she was immediately seized with a terrible spasm; became black in the face and frothed at the mouth which she kept constantly moving; eyes wide open and with the pupils much dilated, became instantly dry and glazed; her body was bent backward on the instant but quickly relaxed so she laid nearly flat on the bed. As this paroxysm seemed passing off another came on, throwing her body again into the backward curvature. This paroxysm also gradually relaxed, but before it passed away and in about fifteen minutes from the first seizure another, and more severe one came on; and now her pulse, which had kept full and regular all this time, becoming slow during the paroxysms, and perceptibly rising as they relaxed, suddenly stopped.

